# Medical Cliniques

**Published:** 1868-04

**Authors:** A. M. Carpenter

**Affiliations:** Professor of Institutes and Practice and Clinical Medicine, Medical Department Iowa State University


THE
### IOWA MEDICAL JOURNAL.

Vol. V. *January—April,* 1868. Nos. 2 & 3.

### MEDICAL CLINIQUES.

BY A. M. CARPENTER, M. D.,

Professor of Institutes and Practice and Clinical Medicine, Medical Department Iowa State University.

### *Incomplete Hemiplegia, with Enuresis.—Recovery.*

Case No. 5, Nov. 6, 1867.—*History.*—Wm. J., *aet.* 75, presented himself to the Medical Class, and disclosed the following history:

About ten months ago, felt a sensation of numbness in left arm and leg; attempted to grasp his cane, but failed; was assisted to his feet, but found himself unable to walk; leg was dragged after its fellow in a swinging motion, describing the arc of a circle; was treated with cathartics and gentle friction to the limbs, the use of which he recovered in about two months, sufficient to admit of his walking a mile without much fatigue.

On the 1st of October, 1867, he experienced a recurrence of the hemiplegic symptoms, together with partial paralysis of the sphincter of the bladder.

*Symptoms.*—Partial loss of both the motor and sensory system; appetite good; bowels costive; sleeps well, with the exception of having to rise a "dozen times at night," to void the urine in small quantities. Mental condition not impaired. Never before the above date was he compelled to get up to void the urine. No spinal irritation present. In aged persons relaxation of the vesical sphincter not unfrequently exists, independently of both spinal irritation and cerebral hemorrhage. However, the weight of evidence in this case strongly favors the conclusion that extravasation did exist, though moderate in degree, while the prediction is safe that the patient will yet succumb to fatal paralysis.

*Treatment.*—℞. Co. Cath. Pill No. xij. *Sig.*—Three every alternate night to render bowels soluble. Moderate friction was directed to be practiced upon the leg and arm twice daily, with a coarse towel. For the incontinence, he took the following: ℞. Ext. Belladonna Sol. gr. vi., div. in pill No. 24. *Sig.*—One thrice daily. Nov. 12th, reports much better; bowels soluble; gait steady, better use of limbs; voided urine eight times during the previous night. To discontinue purgative, continue friction and the Belladonna pills as before, with the addition of one pill at night: dose $\frac{1}{2}$ gr. instead of $\frac{1}{4}$ gr., together with the cold water *douche* to the perinium every night until the 15th December, when he again reported that he was not compelled to get up oftener than once at night, that his limbs were "as good as ever," and he was discharged with the injunction to take the following *mist.* as a finale to the treatment: ℞. Tr. Mur. Ferri ℥i., Tr. Cantharis ʒi., *m.* *Sig.*, 25 drops thrice daily diluted.

### *Incontinence of Urine, with Vulvitis.—Recovery.*

CASE No. 14, Nov. 21st, Mrs. M———, *aet.* 50.

*Symptoms.*—Has suffered from inability to retain her water for three years; general health much impaired during that period; appetite fair; tongue coated; bowels confined; to use her own expression, "get up thirty times a night to pass water;" much pain during the act; suffers from pruritus vulvæ —due to the acrid quality of urine; sleep a good deal disturbed; afflicted with frightful dreams occasionally.

Test of urine showed slight acid reaction. The sound being introduced, no calculus was found. Specular examination revealed nothing abnormal. Hyperesthesia of vulva existed in a marked degree, with tumefaction and reddened state of membrane lining the labia; slight excoriations internal to labia majora, and externally consequent upon the friction of the parts in walking; inability to walk without great pain.

*Treatment.*—To take three Co. Cathartic pills at bed time. To allay the itching and soothe the vulvitis, she was directed to bathe the parts in mucilage of flaxseed during the day, and immediately after emptying the bladder, to dry off the parts and apply the following lotion; ℞. Sodæ Biborat, ʒi.; Morph. Sulph. gr. iv.; Aq. Rosa ℥ iv. To relieve the irritation of the viscus and arrest the incontinence, she took gr. *ss.* Ext. Belladonna thrice daily. Nov. 27, reports got up night before thirteen times to void urine. Itching of labia less; sleeps better; appetite improving. To continue treatment. December 15th, reports progress towards recovery; aspect improved; urine alkaline; vulvitis and pruritus disappeared; bowels open; walks several squares daily without pain or discomfort; voids urine four times per night. Feeling well enough to leave the infirmary, she was put upon the Tr. Ferri Mur. gtt. xxx. thrice daily, and discharged.

---

### SURGICAL CLINIQUES.

Medical Department Iowa State University.

